# Novel Plasmid Carrying Mobile Colistin Resistance Gene *mcr-4.3* and Mercury Resistance Genes in Shewanella baltica: Insights into Mobilization of *mcr-4.3* in *Shewanella* Species

**DOI:** 10.1128/spectrum.02037-22

**Published:** 2022-11-14

**Authors:** Nachiket P. Marathe, Francisco Salvà-Serra, Priyank S. Nimje, Edward R. B. Moore

**Affiliations:** a Institute of Marine Research, Bergen, Norway; b Department of Infectious Diseases, Sahlgrenska Academy, University of Gothenburg, Gothenburg, Sweden; c Department of Clinical Microbiology, Culture Collection University of Gothenburg (CCUG), Sahlgrenska University Hospital, Sahlgrenska Academy, University of Gothenburg, Gothenburg, Sweden; d Centre for Antibiotic Resistance Research (CARe), University of Gothenburg, Gothenburg, Sweden; e Microbiology, Department of Biology, University of the Balearic Islands, Palma de Mallorca, Spain; Instituto de Higiene

**Keywords:** Atlantic mackerel, heavy metal resistance, *Shewanella* spp., antibiotic resistance, colistin resistance, *mcr* genes

## Abstract

*Shewanella* species have been identified as progenitors of several clinically important antibiotic resistance genes. The aim of our study was to analyze Shewanella baltica strains isolated from the gut contents of wild Atlantic mackerel (Scomber scombrus) for the presence of both known and novel variants of antibiotic resistance genes (ARGs), using Illumina-based whole-genome sequencing (WGS). Thirty-three S. baltica strains were isolated from Atlantic mackerel collected in the northern North Sea. WGS revealed the presence of several new variants of class C and class D beta-lactamases. Nearly 42% (14/33) of the strains carried the mobile colistin resistance gene *mcr-4.3*. To understand the genetic context of *mcr-4.3*, we determined the complete genome sequence of strain 11FHM2, using a combination of Oxford Nanopore- and Illumina-based sequencing. The complete genome sequence is 5,406,724 bp long, with one contig representing a chromosome of 5,068,880 bp and three contigs representing novel plasmids (pSBP1, 194,145 bp; pSBP2_mcr4, 86,727 bp; and pSBP3, 56,972 bp). Plasmid pSBP2_mcr4 contains the mobile colistin resistance gene *mcr-4.3*, as well as the mercury resistance operon *merRPAT*. Plasmid pSBP1 carries genes encoding resistance against copper, zinc, chromium, and arsenic. Plasmid pSBP3 does not carry any antibiotic or heavy metal resistance genes. Analysis of the flanking region of *mcr-4.3* suggests that a phage integrase may be involved in the mobilization of *mcr-4.3* in *Shewanella* spp. Our results provide insights into the mobile *mcr-4.3* present in *Shewanella* spp. and highlight the importance of the marine environment in the emergence and dissemination of clinically important resistance genes.

**IMPORTANCE** We identified two new plasmids in Shewanella baltica isolated from wild Atlantic mackerel (Scomber scombrus) collected from the northern North Sea, one plasmid carrying the *mcr-4.3* gene for colistin resistance and the operon *merRPAT* for mercury resistance and the other carrying multiple heavy metal resistance genes. The marine environment has been recognized as a source of new resistance genes that are found in human pathogens. Selection pressure from heavy metals is seen in the marine environment, especially associated with human activities, such as waste discharge, mining, and in aquaculture settings. This would help maintain and disseminate these plasmids in the environment. Our study provides insights into the mobilization of colistin resistance genes in *Shewanella* spp. and highlights the importance of the marine environment in the emergence and dissemination of clinically important antibiotic resistance genes.

## OBSERVATION

The rapid rise in resistance against the so-called last-resort antibiotics, such as carbapenems, vancomycin, and colistin, is a global public health challenge (1). Colistin, also known as polymyxin E, belongs to the polymyxin class of antibiotics and is a last-resort antibiotic used to treat infections of multidrug-resistant Gram-negative pathogens (2). Phosphoethanolamine (PEA)-lipid A transferase is a recently discovered mobile colistin resistance gene (*mcr*) that modifies lipid A moieties by the addition of phosphoethanolamine to glucosamine 1(4′)-phosphate in the bacterial lipopolysaccharide, which reduces the affinity of the cell membrane for colistin, thus conferring resistance (3, 4). Mobile colistin resistance protein (MCR) is widespread globally, with several variants of the *mcr* gene discovered thus far (5, 6). Nonmobile *mcr* genes in aquatic bacteria, particularly in certain species of *Shewanella*, have been hypothesized to be progenitors of the *mcr-4* gene (7). In addition to *mcr-4*, *Shewanella* spp. have been reported to be the progenitors for other clinically important genes, such as OXA-48 and *qnrA* (8, 9).

The aim of our study was to analyze Shewanella baltica strains isolated from the gut contents of wild Atlantic mackerel (Scomber scombrus) for the presence of both known and novel variants of antibiotic resistance genes, using whole-genome sequencing (WGS). Here, we describe a novel plasmid in S. baltica strain 11FHM2 that carries the *mcr-4.3* gene and a mercury resistance operon. This is the first report of plasmid-borne *mcr-4.3* in *Shewanella* spp. and the first report of *mcr-4.3* in S. baltica isolated from wild fish. Furthermore, we show the presence of several new variants of class C and class D β-lactamases, as well as a novel *catB*-related chloramphenicol O-acetyltransferase in S. baltica isolates.

Atlantic mackerel (*n* = 20) were collected from the northern North Sea in October 2018 in sterile plastic bags. The fish were dissected, and the gut contents were collected aseptically in sterile 15-mL tubes. Serial dilutions of the individual gut content samples were inoculated onto Mueller-Hinton agar with ampicillin (100 μg/mL) and onto MacConkey agar with ampicillin (100 μg/mL) and incubated at 30 °C for 24 to 48 h. A total of 73 bacterial isolates were obtained and identified, using matrix-assisted laser desorption ionization–time of flight mass spectrometry (MALDI-TOF MS) (Bruker Daltonics, Germany). A total of 33 isolates were identified as S. baltica. We sequenced the genomes of these 33 strains using the Nextera DNA Flex library prep kit (Illumina, San Diego, CA, USA) for sequencing library preparation, followed by sequencing on the Illumina MiSeq platform, using 2 × 300-bp chemistry and analysis as described previously (10).

Species identification of the 33 strains as S. baltica was confirmed using the Type Strain Genome Server (TYGS) (11). Analysis of the WGS data showed that many of the strains carried new variants of both class C and class D β-lactamases (with amino acid sequence identities ranging from 96 to 98% to the known variants; see Table S1 in the supplemental material). One strain (11FHM2) carried a novel *catB*-related chloramphenicol O-acetyltransferase with 66.5% amino acid sequence identity to the closest known variant. Interestingly, 14 of the 33 (42%) strains carried the *mcr-4.3* gene. A phylogenetic tree including the MCR variant detected in this study (MCR-4.3), along with other known variants of MCR, is presented in Fig. S1.

In order to understand the genetic context of the *mcr-4.3* gene, we determined the complete, closed genome sequence of S. baltica strain 11FHM2 (carrying *mcr-4.3* and a novel *catB* variant), using an Oxford Nanopore MinION instrument, as described previously (12). The complete genome of strain 11FHM2 was determined to be 5,406,724 bp, with one contig representing a chromosome of 5,068,880 bp and three contigs representing novel plasmids (pSBP1, 194,145 bp; pSBP2_mcr4, 86,727 bp; and pSBP3, 56,972 bp). Plasmid pSBP2_mcr4 carries the *mcr-4.3* gene and a mercury resistance operon, *merRPAT*. Strain 11FHM2 also carries a novel megaplasmid, pSBP1, harboring resistance genes against copper, zinc, arsenic, cadmium, and lead.

**FIG 1:**
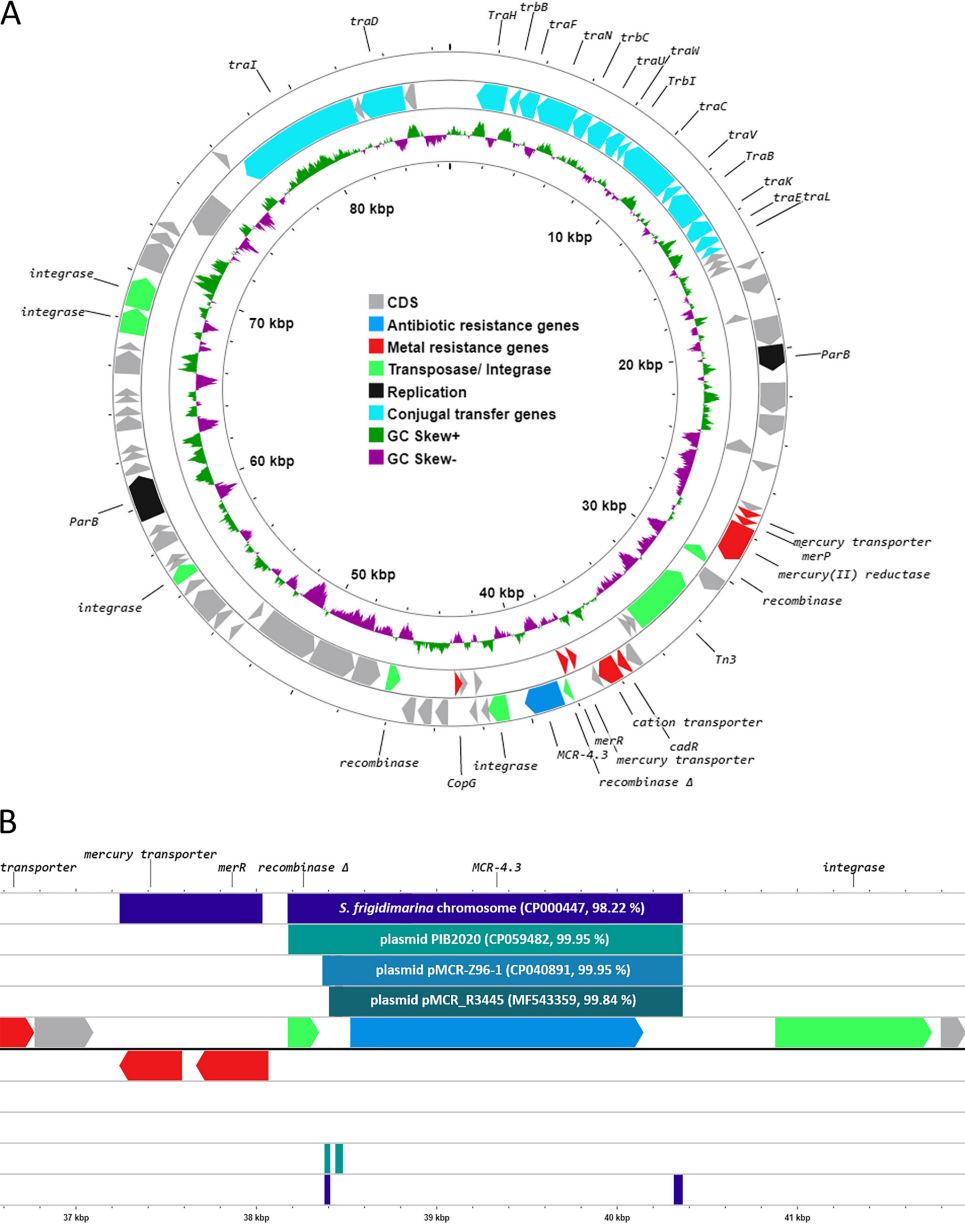
(A) Genetic map of pSBP2_mcr4. (B) Alignment of the region of plasmid pSBP2_mcr4 carrying *mcr-4.3* with Enterobacter kobei strain IB2020 plasmid pIB2020 (GenBank accession number CP059482), Leclercia adecarboxylata strain Z96-1 plasmid pMCR-Z96-1 (CP040891), Salmonella sp. strain R3445 plasmid pMCR_R3445 (MF543359), and Shewanella frigidimarina strain NCIMB 400 (complete genome; CP000447). CDS, coding DNA sequences.

Plasmid pSBP2_mcr4 contains 86 open reading frames (ORFs), with an average GC content of 43.9%. The *mcr-4.3* gene is flanked by a truncated recombinase and integrase (Fig. 1A). Alignment of the DNA fragment containing *mcr-4.3* from plasmid pSBP2_mcr4 (nucleotide positions 38,177 to 40,363) shows that it shares 99.9% nucleotide sequence identity to fragments of several plasmids carrying *mcr-4.3*, as well as 98.2% nucleotide sequence identity to the DNA fragment carrying the *mcr-4.3* gene from the chromosome of the proposed progenitor species, Shewanella frigidimarina (NCIMB 400; GenBank accession number CP000447) (Fig. 1B) (13). The *mcr-4.3* gene in S. frigidimarina NCIMB 400 is flanked by a Tn5044 transposase, while it is flanked by IS*26* and IS*5* transposases in Enterobacter kobei plasmid pIB2020_ColE_MCR (CP059482) and Salmonella sp. plasmid pMCR_R3445 (MF543359), respectively (14, 15). In contrast, in plasmid pSBP2_mcr4, the *mcr-4.3* gene is flanked by an integrase that contains the protein domains Integrase_1 (Pfam PF12835) and Phage_int_SAM_2 (Pfam PF12834). Both domains belong to a family of DNA-binding prophage integrases found in *Proteobacteria*, suggesting a role of phage integrases in the mobilization of *mcr-4.3* in *Shewanella* spp. Our study suggests that the mobilization event of the *mcr-4.3* gene discovered in S. baltica may be independent of the mobilization event for the gene variant found on plasmids in *Enterobacteriaceae*. Having observed that, extensive follow-up studies are needed to ascertain this point (14, 15).

Antibiotic selection pressure is required for maintenance of resistance plasmids. In the absence of selection pressure, i.e., by exposure to antibiotics, plasmids may be lost (16). Other mechanisms of plasmid maintenance include the production of toxin-antitoxin (TA) systems. Toxin-antitoxin systems are important for plasmid maintenance; toxins produced by plasmids kill cells that have lost plasmids during cell division, thus selecting for plasmid-carrying cells (17). Plasmid pSBP2_mcr4 encodes three different type II TA systems of the RelE/ParE type, Phd/YefM family, and HiCAB type. This signifies the potential for maintenance of this plasmid within the marine microbiota.

**FIG 2:**
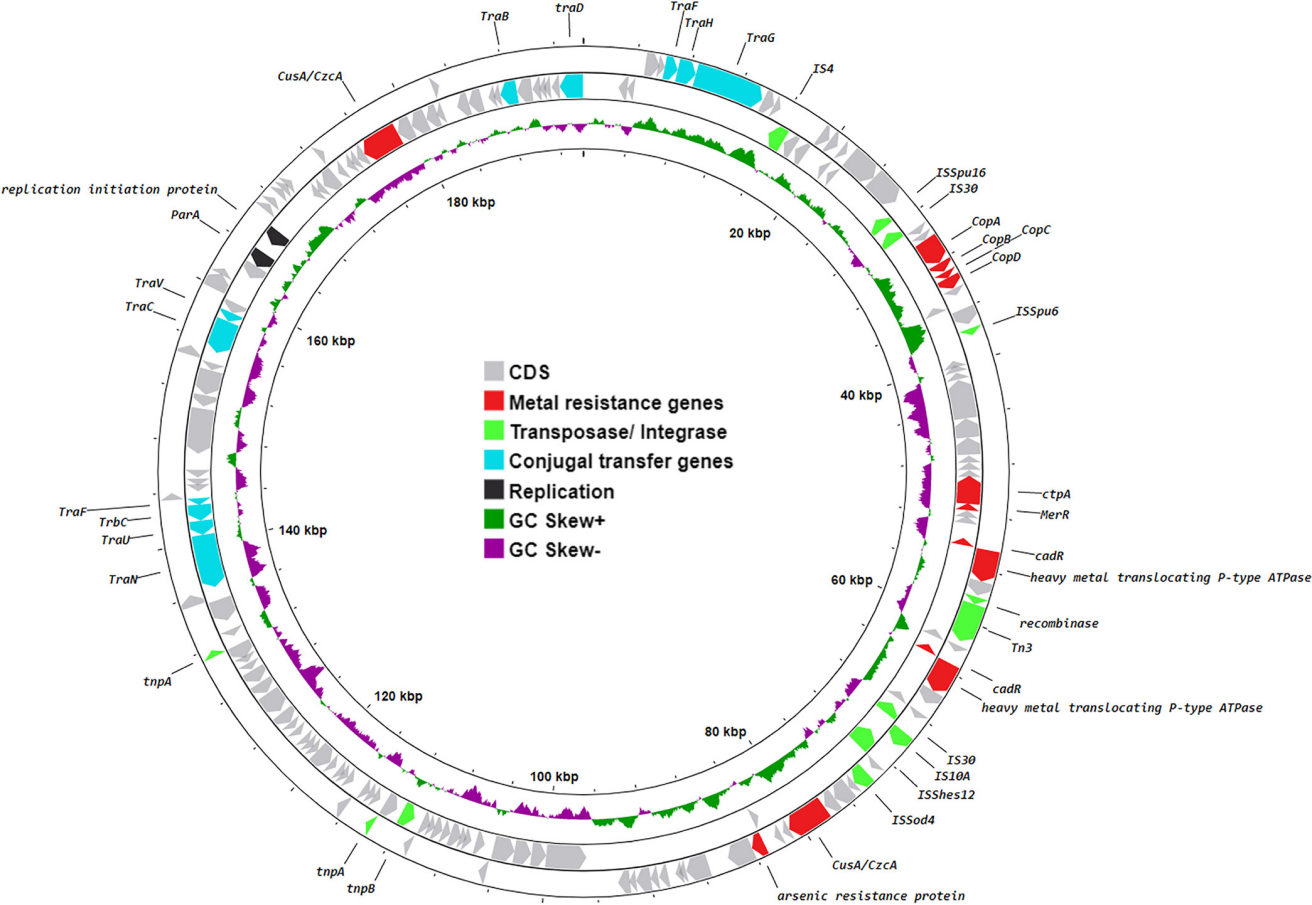
Genetic map of plasmid pSBP1. CDS, coding DNA sequences.

Strain 11FHM2 also carries a megaplasmid, pSBP1 (Fig. 2). Although this plasmid does not encode any antibiotic resistance genes, it does carry multiple heavy metal resistance genes, such as *copABCD*, copper-translocating P-type ATPase, copper oxidase, copper resistance protein NlpE, copper chaperone PCu(A) chromate efflux transporter, CusA/CzcA family heavy metal efflux RND, and arsenic resistance protein. Both plasmids, pSBP1 and pSBP2_mcr4, carry multiple conjugal transfer genes, indicating that these plasmids are potentially conjugative. Marine environments, especially around Norway, have heavy metal selection pressure from the presence of zinc and copper due to mining, as well as the use of heavy metals in aquaculture and antifouling paints (18, 19). These selection pressures may aid in the maintenance of these resistance-carrying plasmids in the marine environment.

The genus *Shewanella* is prevalent in the marine environment and includes some species that are opportunistic fish and human pathogens (20, 21). Due to their ability to grow in cold temperatures, *Shewanella* species, including S. baltica, are commonly found as spoilage bacteria in cold-stored seafood products of salmon and shrimps (22, 23). The presence of a clinically important mobile colistin resistance gene on plasmids in these bacteria pose a risk of not only food spoilage but also transfer of such resistance genes to the human microbiota/pathogens through seafood (24).

Our findings demonstrate the presence of a novel plasmid carrying *mcr-4.3* in S. baltica from wild Atlantic mackerel from the northern North Sea. An untypeable plasmid coharboring the *mcr-4.3* gene and mercury resistance genes was identified. Our study highlights the importance of the marine environment in the emergence and dissemination of important antibiotic resistance genes and the need for environmental monitoring of antimicrobial resistance.

### Data availability.

The complete genome sequence of *S. baltica* strain 11FHM2 has been deposited in GenBank under the accession numbers CP051529 (chromosome), CP051530 (plasmid pSBP1), CP051531 (plasmid pSBP2_mcr4), CP051532 (plasmid pSBP3), respectively. The draft genome sequences of the other strains are deposited in GenBank under the following accession numbers JAAHCG000000000, JAAHCI000000000, JAAHCQ000000000, JAAHCS000000000, JAAHCT000000000, JAAHCU000000000, JAAHCV000000000, JAAHCY000000000, JAAHDA000000000, JAAHDB000000000, JAAHDC000000000, JAAHDD000000000, JAAHDK000000000, JAAHDT000000000, JAAHDZ000000000, JAAHEE000000000, JAAHEI000000000, JAAHEJ000000000, JAAHEK000000000, JAAHEL000000000, JAAHEM000000000, JAAHER000000000, JAAHEU000000000, JAAHEV000000000, JAAHEW000000000, JAAHEX000000000, JAAHEY000000000, JAAHEZ000000000, JAAHFA000000000, JAAHFB000000000, JAAHFC000000000, JAAHCM000000000.
